# Growth and health of juvenile barramundi (*Lates calcarifer*) challenged with DO hypoxia after feeding various inclusions of germinated, fermented and untreated peanut meals

**DOI:** 10.1371/journal.pone.0232278

**Published:** 2020-04-30

**Authors:** Binh Van Vo, Muhammad A. B. Siddik, Md. Reaz Chaklader, Ravi Fotedar, Ashfaqun Nahar, Md. Javed Foysal, Dien Phan Bui, Huy Quang Nguyen

**Affiliations:** 1 Research Institute for Aquaculture No. 1, Hanoi, Viet Nam; 2 Department of Fisheries Biology and Genetics, Patuakhali Science and Technology University, Patuakhali, Bangladesh; 3 School of Molecular and Life Sciences, Curtin University, Bentley, WA, Australia; 4 Department of Marine Fisheries and Oceanography, Patuakhali Science and Technology University, Patuakhali, Bangladesh; 5 Department of Genetic Engineering and Biotechnology, Shahjalal University of Science and Technology, Sylhet, Bangladesh; National Cheng Kung University, TAIWAN

## Abstract

Peanut (*Arachis hypogaea*) is mainly grown for oil extraction and the remaining oil-free seed referred as peanut meal (PM) leaves with high protein content which can be a possible substitute for fishmeal in aqua-diets. This study evaluates the suitability of three types of processed peanut seeds, namely untreated PM (UPM), fermented PM (FPM), and germinated PM (GPM) from peanut seeds to replace fishmeal in barramundi (*Lates calcarifer*) diets cultured under a commercial production environment. Nine formulated diets having 3 inclusion levels from the 3 different peanuts (15%, 30% and 60% fishmeal replacement) were evaluated against a control without PM. The performance of various types and levels of PMs was assessed by examining the growth, gut and liver condition and survival of fish after eight weeks of feeding the test diets. The immunological responses of juvenile barramundi were assessed by exposing the fish to the hypoxic conditions for 4 hours. The results showed that fermentation and germination significantly (P<0.05) reduced the tannins and alkaloid contents in the PMs. The fish fed 15% GPM diet grew faster and had higher survival than fish fed control diet, while fish fed diet including 60% GPM showed a significant reduction in growth and survival, and an increase in food conversion rate (FCR). FPM and UPM at any inclusion levels did not alter the growth, survival and FCR. Histology analysis revealed that fish fed 60% GPM and UPM showed higher amount of lipid droplets in liver, myodigeneration in fish muscle and a decrease number of acidic mucins in distal gut compare to all other test diets. Stress caused by reduced dissolved oxygen did not change the sodium, potassium, chlorides and alanine aminotransferase concentrations of plasma of fish fed any diet. However, the stress did increase plasma cortisol significantly (P<0.05) in fish fed 60% GPM, 30% and 60% UPM diets. These results suggest that the PMs can partly replace the fishmeal in juvenile barramundi diet and the processing further improves the PMs quality by reducing its antinutritional factors which in turn can increase either its inclusion level in the barramundi diets or improved growth and health status of the species.

## Introduction

The increase in fish production through aquaculture has been accompanied by rapid growth of aquafeed production. While fishmeal, a primary protein source in aquafeed, has become limited and expensive, the plant-derived protein ingredients as potential alternatives to fishmeal have gained recognition [1, 2]. A wide range of products from oilseeds, legumes and cereal grains have been tested to determine their optimal inclusion levels to replace fishmeal in fish diets [3–6]. At a higher plant-derived protein inclusion level, the host fish generally show reduced growth and/or altered physiological functions such as significant decrease in plasma protein and increased neutrophil counts as seen in rainbow trout (*Oncorhynchus mykiss*) [6] or increased serum lysozyme as seen in meagre (*Argyrosomus regius*) [7].

Barramundi (*Lates calcarifer*), a carnivore fish is an important cultured species in Australia and Southeast Asia [8]. Reducing production cost by using plant-derived protein to replace fishmeal in the diet is a step forward for the sustainable aquaculture development of the species. A number of studies on barramundi, using protein sources originated from soybean meal [9], canola and wheat gluten [10] and lupins [5, 11] have been published. These studies reported that at a low dietary inclusion levels of plant-derived protein, growth performance of the target fish is not affected, but if higher inclusion level is needs to be used there is a requirement to further bioprocess the selected plant protein source [11].

The failure to use higher inclusion level is due to the presence of higher levels of undesirable antinutritional factors (ANFs) [12] that adversely influence the fish growth and health status of fish. Legumes such as lupins and soybeans contain large amounts of ANFs as soluble and insoluble non-starch polysaccharides, oligosaccharides, phytates, and tannins. All these ANFs negatively affect the fish by decreasing its digestibility [13], palatability and absorption of amino acids [14] and increasing lipid deposition in the liver. The pre-treatments of plant-derived protein are widely practiced to reduce the adverse effects of ANFs and increase the quality of the ingredients [1]. Bio-processed methods such as fermentation and germination have proven to decrease ANFs and increase the bioactive compounds [11, 15], thereby have led to increased dietary inclusion levels, enhanced growth performance and health of the target species.

Peanut (*Arachis hypogaea*), a worldwide important crop, ranks second in term of cropped area after rapeseed (FAS USDA web 2013). Most peanuts are used for oil extraction for human consumption [16], and the rest, peanut meal (PM) as a rich protein source can be an ideal ingredient in aqua-feed. This PM, as other plant rich protein sources, contains ANFs such as tannins, trypsin and amylase inhibitors [17], which can have adverse effects on the fish. Methods to reduce ANFs in peanut by germination, roasting, and a combination of roasting and germinating were shown to improve the peanut quality by [17] but PM were never tried as protein sources in aqua-feed.

In many cases, observing only fish growth and mortality are not enough to evaluate an alternative to fishmeal, the biochemical indices, gut morphology and liver condition could be more reliable indicators assessing fish health. Thus, this study assessed the processed peanut meals (PMs) as a potential protein source to replace fishmeal in juvenile barramundi diets by evaluating growth, gut and liver health, and immunological indices of fish challenging to an acute hypoxic condition.

## Materials and methods

### Ethic approval

The study protocols and design were approved by the Ethics Committee of Curtin University, Australia (Approval No. AEC_2014_14/25). Fish handling activities and all samples were extracted based on the Australian code for the care and use of animals for scientific purposes.

### Peanut meal and bio-processing

Whole peeled peanut seeds of 40 kg were purchased from a local store in Hungloc-Nghean, Vietnam and were divided into two equivalent portions. One portion was used for germination while the other for oil extraction. The extraction of oil was performed by steaming the seed at 90°C for 2 hours before grinding to about 50 μm; then the grounded seed was expressed mechanically (6YL165A, China) until most of the oil was extracted. Oil extracted product was then ground again to 200μm size that was ready to be used as an alternative ingredient to fishmeal. This oil free peanut was termed as untreated peanut meal (UPM).

Half of the UPM was further fermented by *Lactobacilli* spp. as described in previous study using lupin [11]. Cultures of *L*. *acidophilus*, *L*. *aporogenes* and *L*. *kefir*, were obtained from a commercial product BIOLAC, BIOPHARCO, Vietnam, and mass incubated in MRS broth medium (Merck KgaA Germany) containing polysorbate, acetate, magnesium and manganese. To each 1000 ml of distilled water was added 55 g MRS broth and 250 ml soy extract. The combination was autoclaved at 121°C for 15 minutes prior to culturing of *Lactobacilli* spp. The incubation was carried out in a black glass jar with minimum oxygen at 37°C for 24 hours in a refrigerated incubator (Scientifica, VELP). After incubation samples were collected to check if bacterial density was reached to > 10^7^ CFU ml^-1^, while the remaining part was mixed with autoclaved UPM in a plastic bag where commercial N_2_ gas from Hai Duong Gas Company, Vietnam, was filled to enhance anaerobic conditions. The UPM fermentation was conducted at 37°C for 72 hours. The final fermented UPM product was term as fermented peanut meal (FPM)

Germination was performed at room temperature of 25–27°C and 90% humidity. The whole peanut seed was rinsed under dark conditions in a 70-L aluminium container by fresh 37°C warm water for 12 hours. After that, the water in the container was drained out. Germination took place in the same container where the seed were soaked with water at 33–37°C in every 6 hours. After germination, seed with about 2 mm sprouts were selected for oil extraction. The oil extraction and grinding were performed as outlined earlier in this section to become germinated peanut meal (GPM). Finally, the 200g samples from UPM, FPM and GPM were collected to analyse the tannins and alkaloids levels in them.

### Diets preparation

Diets were formulated based on the nutritional composition of the raw ingredients to meet 45% protein and 13% lipid levels (Table 1). All ingredients, except the peanut were obtained from Speciality Feeds, 3150 Great Eastern Highway, Glen Forrest, WA 6071, Australia. Mycotoxin binder, mould inhibitor and stay C were purchased from Feed Company, Ca Mau, Vietnam. Ten diets from various inclusion levels (0%, 15%, 30%, and 60%) of UPM and FPM, and GPM replacing the fishmeal were prepared and labelled as, 15FPM, 30FPM, 60FPM (FPM based diets), 15GPM, 30GPM, 60GPM (GPM based diets), 15UPM, 30UPM, 60UPM (UPM based diets). The diet contained no PM having 630 g kg^-1^ fishmeal was used as a control diet. Diets were processed by addition of water to about 35% mash dry weight with well mixing to form the dough. This dough was then screw pelleted by a laboratory pelletiser to 1.2–2 mm pellets. These moist pellets were oven dried at 60°C for 12 hours followed by cooling to room temperature before storing at– 20°C till further use.

**Table 1:** Composition of control and test diets (FPM, Fermented Peanut Meal, GPM, Germinated Peanut Meal; UPM, Untreated Peanut Meal).

Ingredient	Control	15UPM	30UPM	60UPM	15FPM	30FPM	60FPM	15GPM	30GPM	60GPM
^a^FM	63.00	53.55	44.10	25.20	53.55	44.10	25.20	53.55	44.10	25.20
^b^UPM		9.45	18.90	37.80						
^c^FPM					9.45	18.90	37.80			
^d^GPM								9.45	18.90	37.80
^e^FO	9.20	9.10	9.00	8.80	9.10	9.00	8.80	9.10	9.00	8.80
^f^WF	12.90	12.90	12.90	10.18	12.90	12.90	10.18	12.90	12.90	10.18
^g^BM	3.00	3.00	3.00	3.00	3.00	3.00	3.00	3.00	3.00	3.00
^h^CM	6.98	4.58	2.28		4.58	2.28		4.58	2.28	
^i^CG	3.00	5.50	7.90	13.10	5.50	7.90	13.10	5.50	7.90	13.10
Soy lecithin	1.00	1.00	1.00	1.00	1.00	1.00	1.00	1.00	1.00	1.00
Mycotoxin binder	0.05	0.05	0.05	0.05	0.05	0.05	0.05	0.05	0.05	0.05
Vitamin Premix F2	0.50	0.50	0.50	0.50	0.50	0.50	0.50	0.50	0.50	0.50
Mineral Premix F1	0.25	0.25	0.25	0.25	0.25	0.25	0.25	0.25	0.25	0.25
Mold Inhibitor	0.05	0.05	0.05	0.05	0.05	0.05	0.05	0.05	0.05	0.05
Stay C—35%	0.03	0.03	0.03	0.03	0.03	0.03	0.03	0.03	0.03	0.03
Total	100	100	100	100	100	100	100	100	100	100
Diet protein	45.53	45.58	45.55	45.58	45.61	45.55	45.58	45.60	45.55	45.59
Diet lipid	13.71	13.73	13.76	13.70	13.73	13.76	13.78	13.73	13.76	13.79

^***a***^Fishmeal (FM): Dry matter (92.30%), Crude protein (60.10%) and Crude lipid (11.0%)

^***b***^Untreated peanut meal (UPM): Dry matter (88.20%), Crude protein (48.0%) Crude lipid (11.0%), Carbohydrate (9.80%) and Fibre (7.20%)

^***c***^ Fermented peanut meal **(**FPM**):** Dry matter (89.42%), Crude protein (47.30%) Crude lipid (10.35%), Carbohydrate (7.40%) and Fibre (7.15%)

^***d***^Germinated peanut meal (GPM): Dry matter (92.0%), Crude protein (44.0%) Crude lipid (6.90%), Carbohydrate (6.60%) and Fibre (8.70%)

^e^Fish oil (FO): Derived from salmon, Dry matter (95.0%), and Crude lipid (99.0%)

^***f***^Wheat flour (WH): Dry matter (93.0%), Crude protein (12.0%) Crude lipid (1.80%), Carbohydrate (62.70%) and Fibre (1.0%)

^g^Blood meal (BM): Dry matter (94.0%), Crude protein (90.0%) Crude lipid (1.20%)

^***h***^Cassava meal (CM): Dry matter (91.0%), Crude protein (2.0%), Carbohydrate (62.0%) and Fibre (2.0%)

^***i***^Corn gluten (CG): Dry matter (92.0%), Crude protein (77.0%) Crude lipid (2.10%), Carbohydrate (15.80%) and Fibre (1.20%)

### Experimental design

A 4x3 factorial design was used where factors were 4 inclusion levels (0%, 15%, 30% and 60%) and 3 types of PM (FPM, GPM and UPM). Juvenile barramundi were obtained from Northern National Marine Broodstock Centre, Vietnam and shipped to National Freshwater Breeding Centre (NBC), Vietnam where the juveniles were raised until they were adapted to salinity of 0 ppt. The fish were then acclimated for two weeks and fed Uni-President-Vietnam feed (45% protein, 12% fat). These fish were then graded, and those within the weight range of 6.0–6.5 g each were selected randomly into thirty tanks of 3.5 m^***3***^ each. The flow-through culture systems were set up in an open outdoor shed with a roof to protect from rain and direct sunlight. The natural temperature and photoperiod ranged between 28–31^***°***^C and 12 hours light respectively. After acclimation the experimental fish were fed for eight weeks with the ten different pre-designed diets (Table 2). Every diet was fed in triplicate and three times daily (8 am, 12 am and 4 pm) till satiation which reached within 20 minutes. The number of dead fish were recorded every day to calculate mortality rates. After eight weeks of feeding test, all fish in individual tank were weighted to determine growth rate and feed conversion ratios.

**Table 2:** Tannins and alkaloid concentrations in peanut meal processed by different methods.

	FPM	GPM	UPM
Tannins (%)	0.27^c^±0.03	0.48^b^±0.02	0.69^a^±0.02
Alkaloid (%)	4.64^b^±0.33	1.22^c^±0.06	8.49^a^±0.26

Data are expressed in mean and standard error of the mean of 3 samples. Within rows, values followed by different letters are significantly different (P<0.05, one way ANOVA with Dunnetts multiple comparisons test). FPM, fermented peanut meal, GPM, germinated peanut meal; UPM, untreated peanut meal.

The challenge test to depict acute exposure to reduced dissolved oxygen (DO) was performed to the fish after eight weeks of feeding with 10 test diets. To perform the acute challenge test, five fish were randomly collected from every tanks and kept in closed 5-L plastic bags of freshwater where DO was reduced from 5 mg L^***-1***^ to 2 ± 0.2 mg L^***-1***^ for 4 hours. The water used to DO exhaust was taken from the same tank that the fish were collected. The reduction of DO was carried out by pumping continuously pure N_***2***_ gas, purchased from Hai Duong Gas Company (Vietnam) into the water. DO was measured by HI9146 Portable Dissolved Oxygen Meter (HANNA Instruments).

### Fish handling and sampling

Blood samples were carried out under an application of 2-phenoxyethanol with a dose of 0.2 ml L^-1^; fish were killed with a dose of 0.4 ml L^-1^ after blood sampling [18]. Fish blood samples were collected at the end of feeding trial and after the fish were challenged by reduced DO. In every tank, one blood sample was collected from five fish using a 1-mL syringe and an 18G needle via the caudal tail vein. Blood was stored in single Eppendorf tube (Eppendorf, North Ryde, NSW, Australia). The tubes were then centrifuged at 1000 g for 5 min to settle the erythrocytes and then plasma was transferred to a new Eppendorf tube prior to freezing and then sent for plasma analysis to MELATEC hospital, Hanoi, Vietnam for chemical analyses.

### Sample analyses

Tannins and alkaloids in all types of PM were analysed in National Institute for Food Control, Hanoi, Vietnam. All blood samples were analysed at Laboratory of Melatec Hospital, Hanoi, Vietnam. Blood chemical parameters consisted of sodium, potassium, chloride, alanine aminotransferase (ALT), cortisol, and glucose were analysed as described by Suski, Killen [19]. The plasma concentration of sodium and potassium were measured by using a flame photometer (Model 2655–00) while plasma chloride concentrations were determined by using chloridometer (Model 4435000). The plasma concentration of cortisol was measured by competitive protein binding using a commercially available kit (Coat-a-Count, Diagnostic Products Corporation, Los Angeles, CA, USA). The plasma ALT and glucose concentrations were qualified enzymatically following the methods of Lowry and Passonneau [20] in a 96-well microplate read with commercially available spectrophotometer (Specitra Max Plus 384, Model 05362, USA). The growth performance indices such as SGR, FCR and survival were calculated using the following formula.

$$Specific\;growth\;rate\;(SGR,\%/day)=\left[\frac{ln\;(final\;body\;weight)-ln\;(pooled\;initial\;body\;weight)}{days}\right]\times 100$$

$$Feed\;conversion\;ratio\;(FCR)=\left[\frac{dry\;feed\;fed}{wet\;weight\;gain}\right]$$

$$Survival\;(\%)=\left[\frac{number\;of\;final\;fish}{number\;of\;initial\;fish}\right]\times 100$$

### Histopathology and histomorphology

For hepatic, muscle and intestine histological evaluation, three fish in each treatment were randomly euthanized with AQUI-S at 175 mg/L and dissected to excise liver. Liver and muscle tissues were immediately fixed in 10% buffered formalin prior to dehydrate in series of ethanol and equilibrate in xylene. Then the tissues were embedded in paraffin wax, sectioned at approximately 5 μm using a rotary microtome machine and stained with hematoxylin and eosin (H&E) according to standard histological procedures. Photographs of histological slides were scanned under a light microscope (BX40F4, Olympus, Tokyo, Japan). Intestinal sections were stained with Alcian Blue (AB) pH 2.5 to visualize acidic mucins which were counted from ten highest intact villi under a light microscope (BX40F4, Olympus, Tokyo, Japan), following the methods of Sewaka, Trullas [21] and Chaklader, Siddik [22].

### Statistical analysis

The data were analysed using IBM SPSS for Windows version 25 at Curtin University, Australia and Stata SE 12, USA with the results expressed as the means and standard errors of the mean (SEM). One-way ANOVA was performed to compare ANFs between processed peanut samples and growth performance indices. Two-way ANOVA using general linear model was performed to compare effects of various inclusion levels and peanut types as well as their interaction. Levels of significance were set at P<0.05.

## Results

### Effect of bio-processing of peanut on ANFs

**Fig 1:**
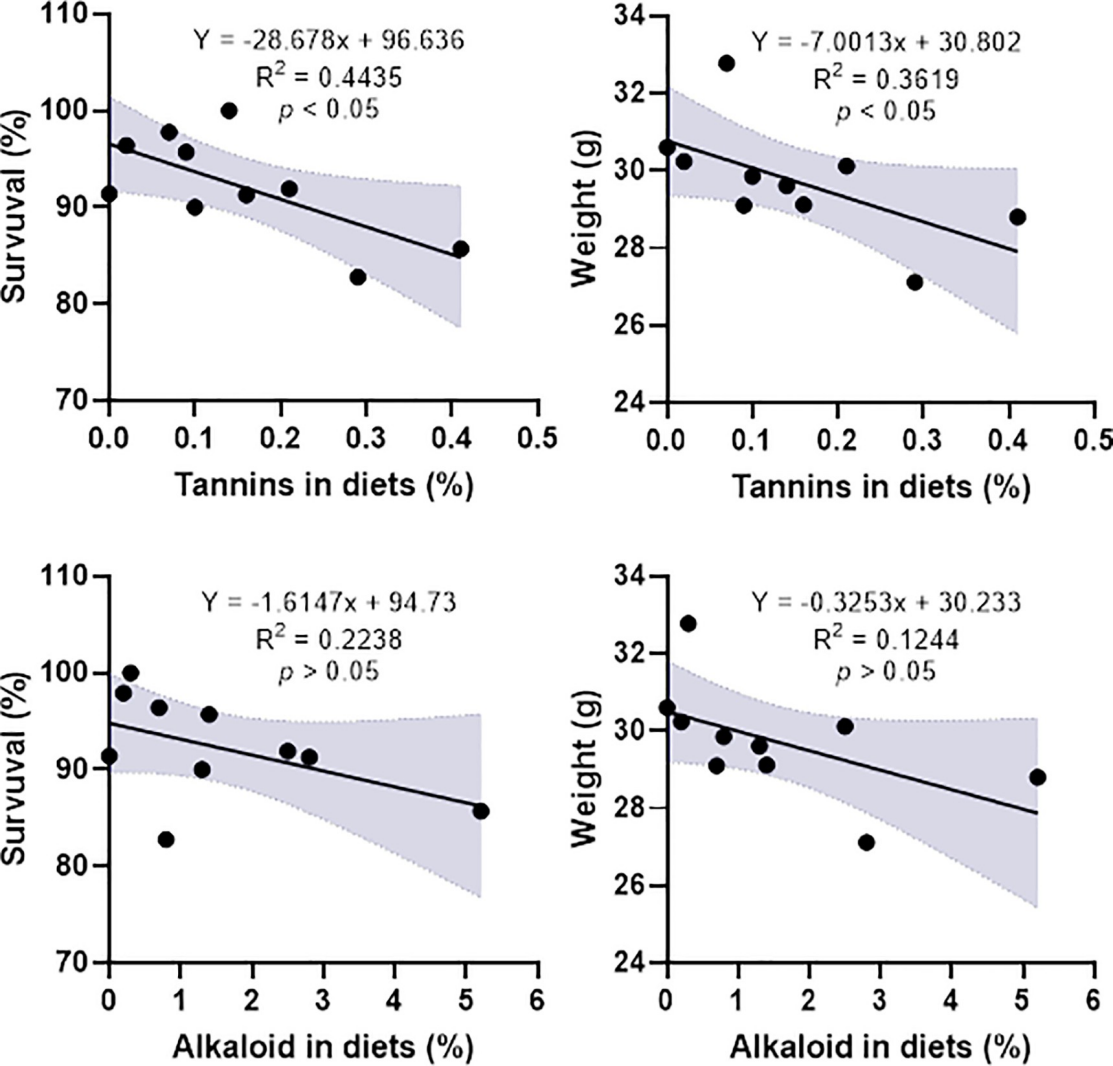
Regression correlation between concentrations of anti-nutritional factors (tannin and alkaloid) in diets and growth and survival of juvenile barramundi.

There were significant differences in ANFs profiles among FPM, GPM and UPM (Table 2). Fermentation and germination resulted in lower concentration (P<0.05) of tannins and alkaloids than the physical oil extraction (UPM). The tannins concentration was significantly reduced at higher rate by fermentation method than that by germination method. In contrast, alkaloid concentration was significantly decreased (P<0.05) by germination than by fermentation. The study also revealed a negative correlation between tannins concentrations in diets with growth and survival rates of fish (Fig 1).

### Growth and survival

The growth performance of juvenile barramundi fed various levels of untreated, fermented and germinated PMs are presented in Table 3. At all inclusion levels, fish fed FPM and UPM grew as fast as when fed control diet, reaching an average weight of 29–32 g. Fish fed 15GPM diet resulted in the highest growth while significantly lowest growth was observed in fish fed 60GPM diet. Fish fed 60GPM diet also grew significantly slower than the fish fed control and all other test diets. SGR showed similar trend as revealed in FBG. Regardless of the types of PM, the growth of fish fed 15% and 30% PMs inclusion levels diets was significant higher than the fish fed 60% PMs diet. An inclusion of 60% PMs in diets resulted in reduced growth compared to control diet. However, when inclusion levels were ignored, different types of bioprocessed PMs included in diets resulted in the same fish growth, feed conversion ratio and fish survival as control diet (Table 4). Feed conversion ratio (FCR) varied from 0.87 to 1.44. Feeding with diets containing FPM and UPM resulted in FCR values of 1.0 or less at all inclusion levels with no significant differences. GPM included in diets, however, resulted in higher variations of FCR values (P<0.05). 60GPM diet resulted in a high FCR value of 1.44, while, 30GPM provided the lowest FCR values (0.87) than control and other test diets.

**Table 3:** Final Body Weight (FBG), Specific Growth Rate (SGR), Feed Conversion Ratio (FCR) and survival of juvenile barramundi fed untreated, fermented and germinated peanut meals at various inclusion levels.

Diets	FBW (g)	SGR (%/day)	FCR	Survival (%)
15FPM	30.24^a^±0.40	2.89^a^±0.21	0.92^b^±0.05	96.40^ab^±3.6
30FPM	29.15^bc^±1.26	2.81^bc^±0.95	1.00^b^±0.04	95.70^ab^±2.5
60FPM	29.12^bc^±0.37	2.81^bc^±0.14	1.03^b^±0.01	91.50^ab^±4.3
15GPM	32.78^a^±2.27	3.02^a^±0.29	1.09^b^±0.09	97.80^a^±1.1
30GPM	29.61^b^±0.60	2.84^b^±0.08	0.87^b^±0.04	100.00^a^±0.0
60GPM	27.12^c^±0.79	2.68^c^±0.07	1.44^a^±0.04	82.40^b^±3.5
15UPM	29.85^b^±0.79	2.86^b^±0.15	1.06^b^±0.01	90.00^ab^±1.9
30UPM	30.16^ab^±1.30	2.88^ab^±0.76	0.98^b^±0.03	91.90 ^ab^±3.0
60UPM	28.80^bc^±0.73	2.79^bc^±0.68	0.95^b^±0.04	85.70^ab^±2.1
Control	30.60^ab^±2.23	2.91^ab^±0.42	0.96^b^±0.04	91.40^ab^±2.5

Data are expressed in mean and standard error of the mean of 3 samples. Within columns, values followed by the same letter are not significantly different (P>0.05). UPM, untreated peanut meal; FPM, fermented peanut meal; GPM, germinated peanut meal.

**Table 4:** Growth performances of juvenile barramundi at different inclusion levels and types of PM.

	Inclusion levels (%)			Types of processed peanut meal				Two-way ANOVA		
	15	30	60	FPM	GPM	UPM	Control	Inclusion levels	PM types	Interaction
FBW (g)	31.00^a^±0.57	29.60^a^±0.55	28.30^b^±0.44	29.60^a^ ±0.49	29.50^a^ ±0.55	29.80^a^ ±0.83	30.60^a^ ±0.51	*	ns	ns
SGR (%/day)	2.87^a^ ±0.74	2.76^a^±0.42	2.71^b^±0.54	2.73^a^±0.42	2.79^a^±0.62	2.72^a^±0.78	2.91^a^±0.46	*	ns	ns
FCR	1.03±0.04	0.95±0.03	1.14±0.08	1.00±0.02	1.44±0.09	0.99±0.03	0.96±0.04	ns	ns	ns
Survival (%)	94.70^a^±1.59	95.90^a^±1.82	86.60^b^±2.05	89.20^a^ ±1.91	94.50^a^ ±1.76	93.50^a^ ±2.77	91.40^a^±2.47	*	ns	ns

Within rows, values followed by the same letter are not significantly different (Bonferroni test). Two-way ANOVA being set significance at P<0.05). FBW, final body weight; SGR, specific growth rate; FCR, feed conversion ratio; UPM, untreated peanut meal, FPM, fermented peanut meal; GPM, germinated peanut meal.

Survival of fish in all dietary treatments was greater than 90% except for the fish fed diet containing 60% GPM and UPM, where survival rates were 82.8% and 85.7%, respectively. Feeding FPM and UPM diets at different inclusion levels did not result in the different survival rates and were similar to the fish fed the control diet. The diet contained 60% GPM showed significantly higher mortality (11.20% in average) than fish fed diets containing 15% and 30% inclusion levels (Table 3). No mortalities were observed in fish fed 30GPM diet. In terms of growth, survival and FCR, no significant interactions were observed among dietary inclusion levels of PM and PM types (Table 4).

### Effect of diets on biochemical responses

The biochemical responses of juvenile barramundi fed untreated, fermented and germinated peanut meals at various inclusion levels are presented in Table 5. Sodium, potassium and chloride concentrations in plasma of the fish at the end of feeding trial ranged from 143–157 mmol L^-1^, 18–20 mmol L^-1^, and 135–145 mmol L^-1^, respectively. Before exposing fish to hypoxic stress, no interaction was observed both in inclusion levels and types of PMs relating biochemical responses of fish. After low DO stress was induced, the plasma sodium and chloride concentrations decreased (127–148 mmol L^-1^, 117–130 mmol L^-1^, respectively) while plasma potassium increased (19–26 mmol L^-1^). However, these changes in immunological parameters were not significant due to feeding different diets or when exposed to DO reduction. Interaction of DO reduction and diets on the change of these immunological parameters were also not observed.

**Table 5:** Concentration of plasma sodium, potassium, chloride, ALT, cortisol and glucose of juvenile barramundi subjected to oxygen reduction shock (from 5.60 mg/L^-1^ to 2.00 mg/L^-1^) for 4 hours).

Diets		Sodium (mmol/L)	Potassium (mmol/L)	Chloride (mmol/L)	ALT (U/L)	Cortisol (mmol/L)	Glucose (mmol/L)
15FPM	Normal	155.20±1.23	19.69±2.35	142.70±1.32	133.93±7.96	128.86±4.65	8.42±1.75
	After DO reduction	148.07±3.56	23.01±1.56	119.70±0.96	116.90±6.56	121.43±2.36	21.72±2.78
30FPM	Normal	157.27±0.89	19.50±3.25	145.97±0.03	159.23±4.32	128.17±7.96	3.90±0.98
	After DO reduction	138.07±1.36	23.76±1.76	122.30±1.29	101.23±2.35	109.96±2.36	24.59±1.89
60FPM	Normal	146.70±4.63	18.06±3.68	137.60±2.35	157.03±4.96	108.44±6.86	5.66±2.45
	After DO reduction	134.63±3.78	23.51±2.19	117.63±1.95	147.40±5.62	123.80±3.26	26.51±2.95
15GPM	Normal	150.57±1.76	20.00±0.22	143.00±1.02	137.33±3.36	143.36±2.56	3.93±1.27
	After DO reduction	131.70±2.89	19.96±10.26	128.23±1.96	154.97±2.56	152.83±2.98	20.81±4.59
30GPM	Normal	151.53±0.76	19.63±5.62	141.97±1.89	78.70±4.23	177.37±5.68	7.00±3.56
	After DO reduction	141.50±4.89	25.62±2.35	13.40±2.38	103.17±2.95	133.40±3.62	21.69±2.96
60GPM	Normal	143.80±2.69	20.00±3.25	138.93±2.56	233.17±7.36	86.07±2.37	5.05±2.35
	After DO reduction	136.73±3.12	21.61±1.23	126.90±1.94	85.30±2.84	162.03±5.36*	31.82±3.12*
15UPM	Normal	144.10±7.89	18.99±4.95	135.67±4.23	122.80±3.26	106.79±4.36	4.07±1.24
	After DO reduction	136.80±2.89	22.86±2.35	128.57±1.23	83.47±4.25	108.23±2.35	18.79±2.31
30UPM	Normal	153.73±1.56	19.62±7.69	141.70±0.54	149.60±2.56	90.43±1.25	4.94±2.89
	After DO reduction	127.57±0.96	25.00±3.26	121.63±1.98	135.73±3.65	202.53±3.26*	23.54±2.45
60UPM	Normal	154.63±0.12	19.93±2.65	142.93±1.85	133.00±2.35	126.63±1.26	7.04±1.95
	After DO reduction	140.37±2.35	26.57±1.32	124.30±3.75	143.10±1.26	247.00±3.26*	28.56±3.12*
Control	Normal	156.37±0.87	18.84±2.65	143.60±1.23	129.63±3.65	140.30±2.85	5.77±3.26
	After DO reduction	128.97±1.52	21.73±1.35	124.57±2.38	116.60±2.35	121.44±3.11	19.4±1.28
*Before hypoxic condition*							
Inclusion levels		ns	ns	ns	ns	ns	ns
PM types		ns	ns	ns	ns	ns	ns
Interaction		ns	ns	ns	ns	ns	ns
*After hypoxic condition*							
DO reduction		ns	ns	ns	ns	**	*
Test diets		ns	ns	ns	ns	ns	ns
Interaction		ns	ns	ns	ns	ns	ns

An asterisk (*) denotes a statistically significant differences in plasma cortisol level between before and after DO reduction. Values are shown as mean ± standard deviation.

The ALT concentrations of plasma of fish groups fed different diets before and after DO reduction were respectively in the range of 78–159 U L^-1^ and 83–154 U L^-1^. Average levels of ALT concentrations had a trend to decrease for the fish fed diets 15FPM, 30FPM, 60FPM, 60GPM, 15UPM, 30UPM and control while increased for the fish fed diets 15GPM and 30GPM. However, these changes were not significant among fish groups fed with various diets without or with DO reduction. There was also not a significant interaction between diets and the DO reduction induced stress in the change of plasma ALT concentration.

Before juvenile barramundi were subjected to DO reduction, the concentration of cortisol in the plasma was 68–177 mmol L^-1^ with the highest level in fish fed 30GPM diet and the lowest in fish fed 60GPM diet. Exposure to reduced DO significantly increased plasma cortisol level in fish fed diets contained 60% of GPM and UPM, and 30% of UPM. Irrespective of this increase, reduced DO failed to influence any plasma cortisol levels of fish among any feeding groups.

The level of glucose concentrations in plasma of fish fed test diets after 8 weeks was in the range of 4.9–8.4 mmol L^-1^. Under DO reduction stress the fish fed all diets resulted in the significantly four-fold increase in plasma glucose concentration (19–31 mmol L^-1^) than the fish without stress in 60GPM and 60UPMP diets. However, DO reduction had no influence in plasma glucose levels of fish among the feeding groups. Interaction between DO reduction and the test diets on physiological parameters were not observed.

### Histopathology and histochemistry

**Fig 2:**
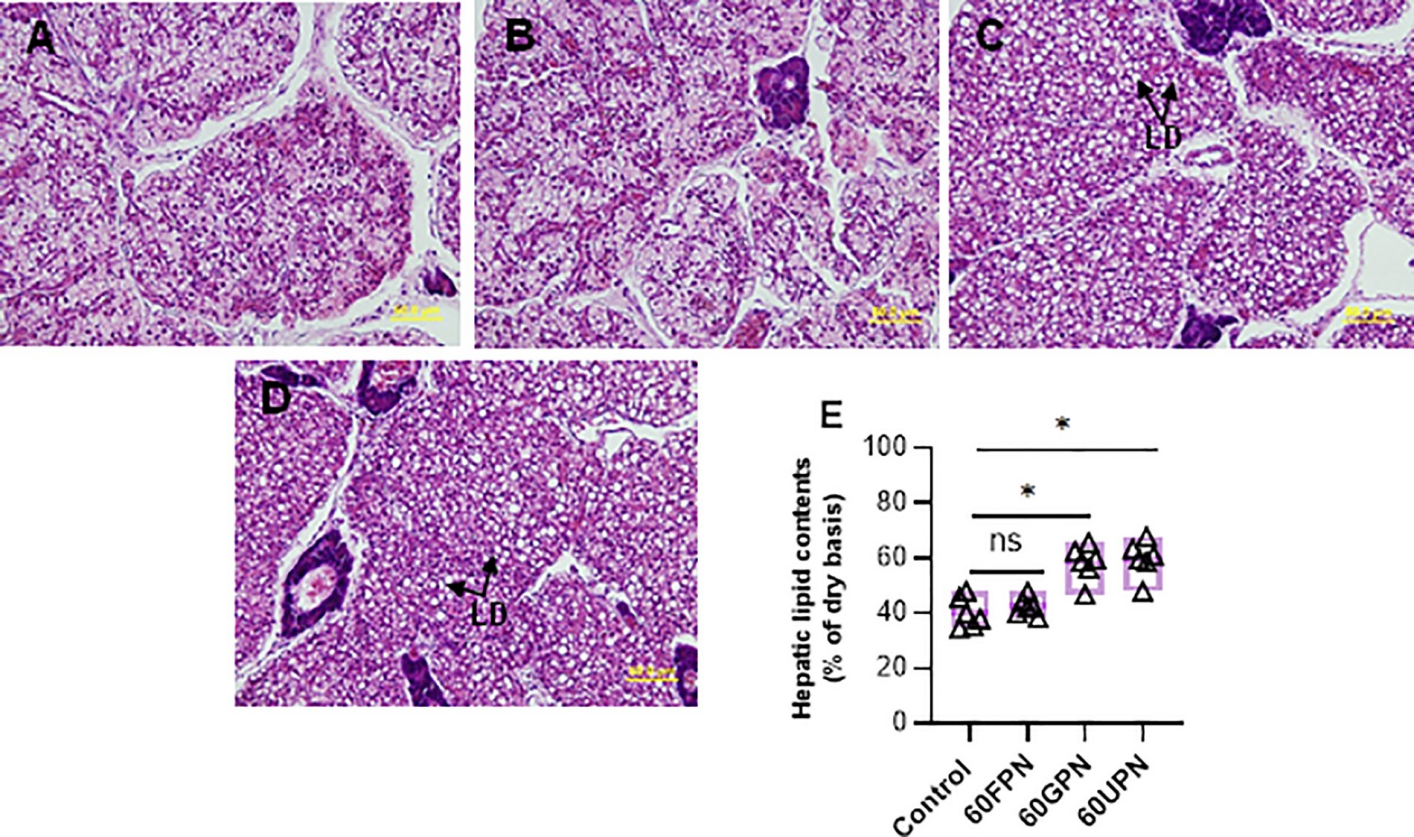
Histological structure of the liver of juvenile barramundi fed control, 60FPN, 60GPN and 60UPN diets (A-D, respectively). Black arrow in liver micrographs indicates large vacuoles in hepatic cells (H&E staining, 40 x magnification, scalebar = 50μm). Variation in the hepatic lipid content in fish fed control, 60FPM, 60GPM and 60UPM after 8 weeks (E). Significant at *P < 0.01 (one-way ANOVA with Dunnetts multiple comparisons test). ns: not significant.

**Fig 3:**
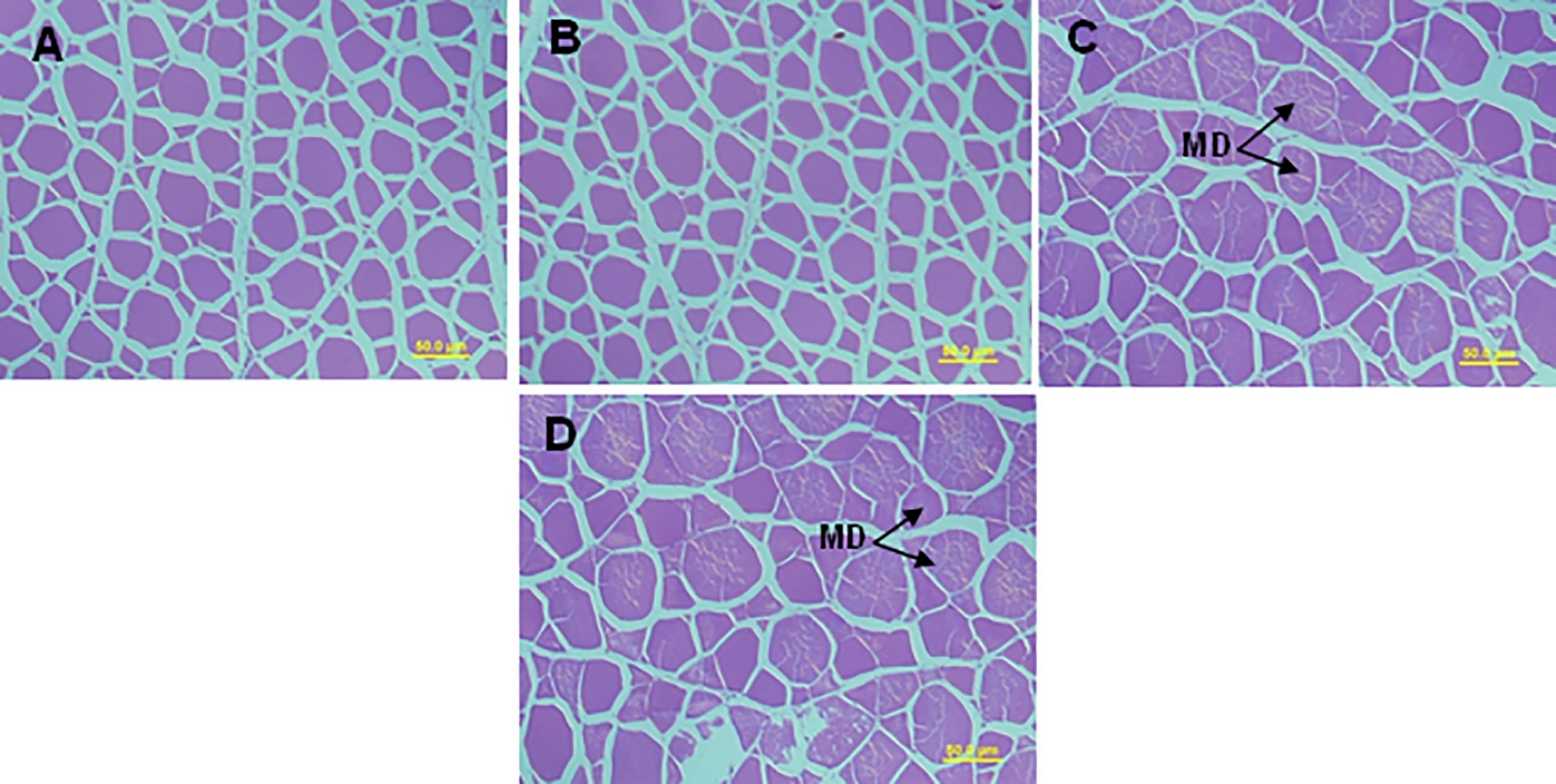
Histological structure of the liver of juvenile barramundi fed control, 60FPM, 60GPM and 60UPM diets (A-D, respectively). Black arrow in muscle micrographs indicates myodigeneration (MD) (H&E staining, 40 x magnification, scalebar = 50μm).

**Fig 4:**
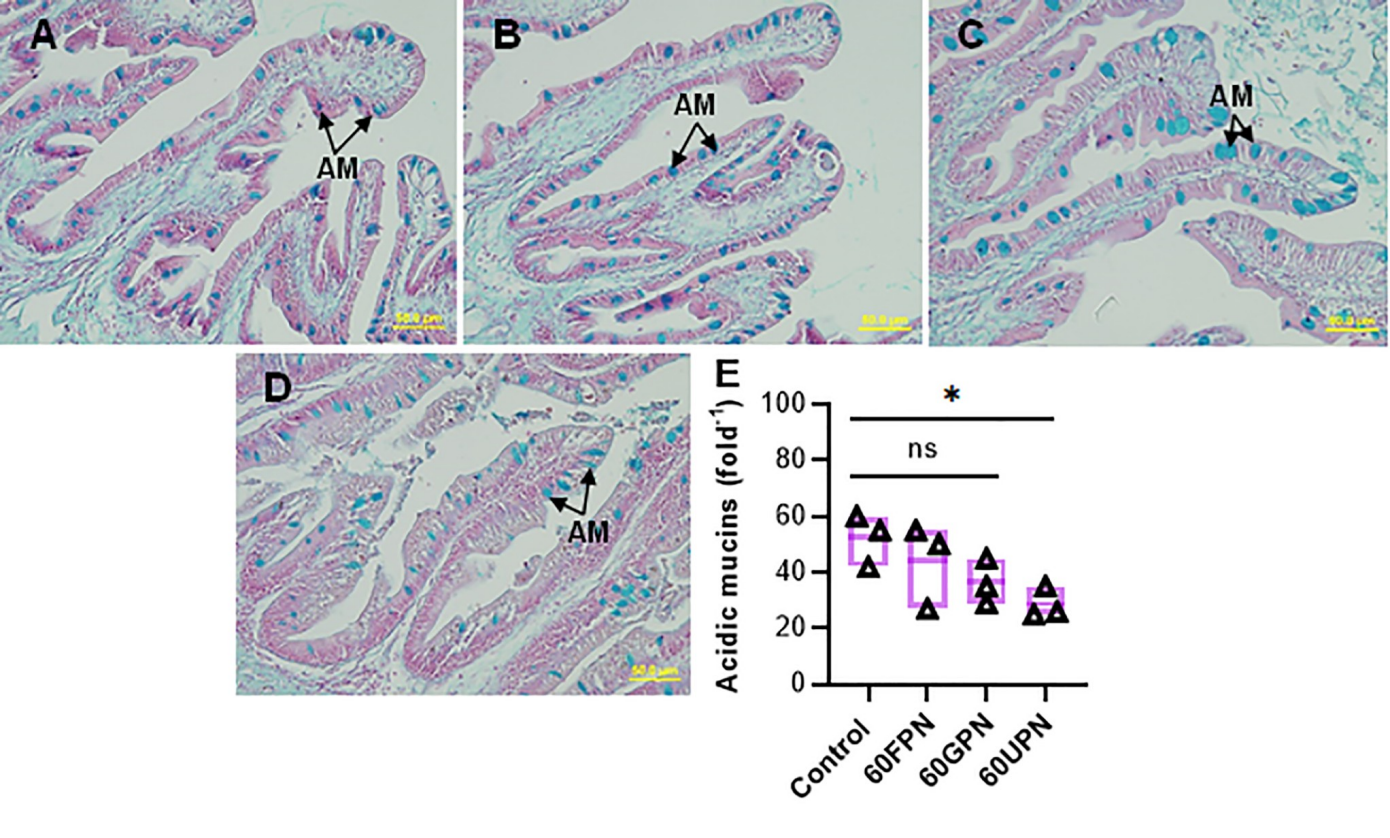
Histological structure of the intestinal section of juvenile barramundi fed control, 60FPN, 60GPN and 60UPN diets (A-D, respectively) (Alcian blue staining, 40 x magnification, scale bar = 50μm). Black arrow indicates acidic mucin cells. Variation in the acidic mucins in the intestine of fish fed control, 60FPM, 60GPM and 60UPM over 8 weeks (E). Significant at *P < 0.01 (one-way ANOVA with Dunnetts multiple comparisons test). ns: not significant.

The hepatic lipid content showed a gradual increase from lower to higher replacement levels of fishmeal by PMs and a significant (P<0.05) increase was found in fish fed 60GPM and 60UPM when compared to control (Fig 2). In accordance with the results of hepatic lipid content, higher lipid accumulation was observed in fish fed 60GPM and 60UPM compared to control and 60FPM, as revealed by hepatic histology (Fig 2). Muscle histology revealed that 60GPM and 60UPM negatively influenced the myotome structure of fish which was attributed by muscle fibre degeneration (Fig 3). The number of acidic mucins cells were decreased significantly in fish fed 60GPM and 60UPM compare to all other dietary groups (Fig 4).

## Discussion

There are several ANFs such as phytic acids tannins, alkaloids, trypsin and amylase inhibitors present in protein rich plants [12] including peanuts [17]. When the PM is used as an ingredient in aqua-feed, ANFs would adversely affect the growth performance of the targeted fish. ANFs are rather stable under heat treatment [23] but can be efficiently removed by fermentation [24]. Lactic acid fermentation has been used to significantly reduce the phytic acid in cereals and sesame seed [25–27]. Fermentation by *Lactobacilli* is able to reduce phytic acids and tannins in sweet lupin (*Lupinus angustifolius*) by 87.04% and 17.64%, respectively [11]. Liang, Han [28] reported that fermentation rather than soaking is more efficient way to decrease the phytic acid of brown rice, *Oryza sativa*.

Pyrrilizidine alkaloids extracted from tansy ragwort (*Senecio jacobaea*) when included in rainbow trout (*Salmon gairdneri*) diet at a concentration of 100mg/kg resulted in severe reduction in growth and survival [29]; and at a lower concentration of 2 mg/kg resulted in hepatic lesions consisting of necrosis, megalocystis, fiber tissue scarring, and occlusion of the hepatic veins. Similarly, tannins included in a diet can reduce palatability and growth in common carp (*Cyprinus carpio*) [30] as they can interfere to decrease the concentration of digestive enzymes as observed in different Indian carps, rohu (*Labeo rohita*), catla (*Catla catla*), and mrigala (*Cirrhinus mrigala*) [31]. In this study, both alkaloids and tannins were reduced by fermentation and germination, of which tannins concentrations in diets had a significantly negative correlation with growth and survival rates (Fig 1).

After 8 weeks of feeding, the barramundi juveniles fed all diets gained greater than 29 g from an initial average of 6.2g at FCR of 1.0 or less, except in fish fed 60GPM diets that resulted in reduced growth of 27 g and increased FCR of 1.44. The reduced growth performance in fish fed higher fishmeal replacement diet of 60GPM may be associated with the deficiency and/or imbalanced rations of certain essential amino acids and fatty acids, and higher variability in biochemical composition in the supplied diet compared to lower fishmeal substitute diet by various PMs. However, there is no published research available that can be used to compare our results with the literature on using PMs as fishmeal replacement diet in aquaculture production. FPM and GPM are also never tested as a diet ingredient for barramundi or any other marine species. In comparison to other plant-derived protein ingredient tested for the same species, our previous investigation [11] reported that sweet fermented lupin (*Lupinus angustifolius*) could increase fishmeal replaced proportion in juvenile barramundi diets which is similar to the current study where 60% fermented peanut inclusion diets resulted in no changes in growth rate and FCR than the control diet.

GPM inclusion in diet seemed to have an effect on palatability of juvenile barramundi. At low inclusion levels of 15% and 30%, fish had a higher feed intake and unchanged FCR than the control diet; but at higher inclusion level of 60%, fish rejected the feed, leading to a very high FCR, as 1.5 fold higher than other test diets. FPM and UPM included in diets did not influence the feed intake or FCR at any inclusion level of PM in diet, the FCR was as low as in control diet.

Supplementary immunological stimulants contained in a feed are believed to increase the fish health and the survival [32]. It is known that during germination, the respiration and synthesis of new cells in the developing embryos of legumes and oil seeds lead to significant changes in biochemical, nutritional and sensory characteristics. Similarly, phenolic content can increase in germinated lupins too [33, 34]. Concentration of vitamins C and E, β-carotene, ferulic and vanillic acids are barely detectable in dry wheat grain but increase significantly after wheat is germinated for 7 days [35]. These antioxidant vitamins are useful for fish growth and increased survival [32, 36, 37]. In our study, the survival of the fish were very high when fed diets containing 15% and 30% of GPM, but got reduced at 60% inclusion level. The higher survival rate in low inclusion levels of GPM based diet could be explained by the presence of antioxidant compounds that are generated in GPM. However, if these compounds are in excess, there could be adverse effects on fish health as shown by Garcia, Pilarski [38] and that could explain the reduced survival in fish fed 60% GPM based diet.

Dietary compositions can influence the biochemical responses of the target species [39]. The inclusion of fermented vegetables increases blood leukocytes of fish [40] similar to enhanced antioxidant activity by feeding the fish germinated lupin [33] which in turn, enhances the resistance to certain stressors. Dietary vitamin C and E induce biochemical and haematological responses, abrogates chlorpyrifos toxicity [32], reduce the impact of infections [37]. These vitamins and antioxidants are believed to be abundant in germinated legumes [15, 33–35]. In the present research, diets including any levels of FPM did not change the plasma glucose. However, 60% GPM or UPM based diets significantly increased the glucose level than the control diet when the fish were exposed to reduced DO levels. Also, plasma cortisol concentrations were significantly increased in fish fed 30% and 60% UPM, supporting the hypothesis that bioprocessed PM provides immunological stimulants into diets and thus improves the fish health and enhances the resistance under stress condition.

The monovalent sodium, potassium and chloride ions are involved in neuromuscular excitability, acid-base balance and osmotic pressure [41]. Potassium plays an important role in nerve conduction, ion balance and gas transport [42]. Ion regulation is energy demanding and disturbances in ionic balance can reduce growth rates and impair swimming performance [43]. On the other hand, elevated levels of ALT, a liver enzymes, in general, signify some form of liver damage or injury [44]. In the present study, there were no significant changes in the concentration of plasma sodium, potassium, chloride and ALT, indicating that the diets and DO reduction stressor did not induce changes in osmotic regulation and liver dysfunctions.

Concentrations of sodium and chloride in plasma in this study were similar to those found by Glencross, Rutherford [10], where seawater juvenile barramundi were fed various plant ingredients and similar levels of these immunological parameters were recorded in fish nursed in saline groundwater fed commercial diet [45]. However, potassium and ALT concentrations in our experiment were five-fold higher than that reported by Glencross, Rutherford [10]. This difference could be explained by the difference in the rearing water salinity where the fish were cultured. In the present study the fish were cultured in freshwater and had plasma potassium levels of 20–25 mmol L^-1^, higher than that in saline ground water (13–14 mmol L^-1^) [45, 46] and in seawater ranging in 3–5 mmol L^-1^ [10]. This demonstrates that plasma potassium and ALT are more likely correlated to culture environments.

The hypothalamus of the stressed fish releases corticotropin-releasing factor and other chemicals in blood circulation which finally activate the release of cortisol by the interrenal tissue. Additionally, the increase in plasma glucose is to cope with the demand of energy as the response of the fish fight against the stressor. Barton, Schreck [47] reported that diets with different levels of lipid can affect the levels of plasma glucose and cortisol. Dietary supplementation of chitosan oligosaccharides at 40mg kg^-1^ can improved phagocytic activities, respiratory burst activities and reduced the level of cortisol when exposure to and challenged by air exposure and bacterial pathogen [48]. In this study, UPM included at medium and high levels (30% and 60%) in diet and under reduced DO stressor, significantly increased the cortisol level, while these fish exhibited normal performances of growth, survival and FCR. Plasma cortisol concentration was also significantly higher in fish fed GPM where they showed reduced growth, increased mortality.

To reduce feed production cost, proportion of fishmeal should be decreased in diets while ensuring a balanced nutritional formulation that provides the fish with good growth performance and health. Protein concentration in processed peanuts is lower than in fishmeal thus high protein ingredients, blood meal and wheat gluten have been used in accordance with levels of the peanuts included into the diets. Meanwhile the cassava meal and wheat flour portions were changed to keep the diets with isonitrogenous and isocaloric. Barramundi used efficiently blood meal and the Inclusion of this ingredient did not affect to the fish growth and FCR [49], but the carbohydrate is less utilized by marine fish [50]. The ingredients’ portion changes in this study did not generate an interaction among test ingredients, suggesting balanced nutrition of diets is acceptable.

Histological observation of liver has been considered as a good indicator to evaluate the nutritional condition of fish, and the most common changes in the liver of fish is the accumulation of fat, which results from impaired protein synthesis or excessive amount of dietary lipid or energy being stored in the hepatic cells unable to oxidise fatty acids [51]. In the present study, fish fed 60% germinated peanut meal or untreated peanut showed obvious changes with high lipid vacuolization in liver which could be due to ANFs like soluble and insoluble non-starch polysaccharides, oligosaccharides, phytates, and tannins. Tannin in present study was significantly higher in GPM and UPM compare to FPM, causing histopathological problems in liver. Utilization of plant meal is also inhibited by the presence of alkaloid and its lower concentration or 2 mg/kg resulted in hepatic lesions consisting of necrosis, megalocystis, fiber tissue scarring, and occlusion of the hepatic veins in rainbow trout, *Salmo gairdneri* [52]. Similarly, higher inclusions of plant proteins increased the hepatocyte vacuolization and hepatic fat deposition in hybrid grouper (*Epinephelus lanceolatus* ♂ × *Epinephelus fuscoguttatus* ♀) [53, 54]. In concomitant with the findings of liver histopathology, necrotic myotome and myodigeneration was present in 60% GPM and UPM. Certain ANFs may hinder the bioavailability of a number of minerals (copper, zinc, iron, magnesium, calcium and selenium) [55, 56], which could affect the muscle structure. For example, Ilham, Fotedar (56) reported that juvenile barramundi, *Lates calcarifer* fed selenium deficient lupin meal showed necrotic muscle, characterized by myodigeneration, while selenium supplemented lupin meal showed no obvious change in muscle and liver. Also, vitamin E deficiency generated myodigeneration in Atlantic salmon, *Salmo salar* [57]. Histopathological changes indicate that irrespective of germination, higher replacement of PMs meal exert negative effects in liver and muscle health of juvenile barramundi. Further study is needed to be done along with the supplementation of minerals in PM.

The complexity of internal architecture of fish intestine is induced by quality and quantity of feed [58]. The number of goblet cells in fish gut is an important indicator for digestion and absorption efficiency [59]. Goblet cells composed of two types of mucins (acid and neutral mucins) which are associated with multiple functions including nutrient absorption, lubrication, trapping and elimination of pathogens [60–62]. Disintegration of intestine such as damaging of intestinal villi and necrotic and degeneration in mucosal epithelium has been widely reported in juvenile turbot, *Scophthalmus maximus* L. [63], Atlantic salmon, *Salmo salar* and Rainbow trout, *Oncorhynchus mykiss* [64, 65] when fishmeal was replaced by plant meal. In line with those findings, the present study found significantly lower number of acidic mucous cell in the intestine of 60GPM and 60UPM fed groups, indicates that higher inclusion of PM impacted the mucosal immunity of juvenile barramundi. The certain components of PM including non-starch polysaccharides, resistant starch, and certain oligosaccharides impacted the intestinal integrity of fish [66] and other animals [67, 68], however, extract of peanut skin containing antioxidants has been suggested to use as functional ingredients in foods [69, 70], which deserve further study.

In conclusion, fermentation of peanut resulted in significant reduction in tannins and alkaloid by 60% and 40% while germination resulted in decline of 86% and 45% of tannins and alkaloids, respectively. The inclusion level of fermented peanut meal in the diet could be increased up to 60%, without compromising the growth performance. However, a reduced growth and higher cortisol level were reported in fish fed 60% germinated peanut meal or untreated peanut. The depressed growth performance in 60% GPM and UPM was further supported by histopathological changes in liver, myodigeneration in muscle and a decrease number of acidic mucins in intestine of juvenile barramundi.
